# Utilizing Telemedicine for Group Visit Provider Encounters: A Feasibility and Acceptability Study

**Published:** 2020-08-02

**Authors:** Tushar A Patel, Craig A Johnston, Victor J Cardenas, Elizabeth M Vaughan

**Affiliations:** 1 School of Health Professions, Baylor College of Medicine, United States; 2 Department of Health and Human Performance, University of Houston, United States; 3 Department of Internal Medicine, University of Texas Medical Branch, United States; 4 Department of Medicine, Baylor College of Medicine, United States

**Keywords:** Minority health, diabetes, telemedicine, chronic disease management, educational research, group visits or shared medical appointments

## Abstract

**Background:**

The value of telemedicine has been underscored during the coronavirus pandemic. Utilizing telemedicine could markedly enhance group visit scalability and sustainability. However, there are limited data demonstrating telemedicine use for group visits.

**Objective:**

To evaluate the feasibility and acceptability of provider encounters conducted via telemedicine in group visits.

**Materials and Methods:**

We conducted a 6-month diabetes group visit program and compared in-person (months 1–3) versus telemedicine (videoconferencing) (months 4–6) patient-provider encounters. Participants completed the Telehealth Usability Questionnaire (TUQ) at 6-months (primary outcome). To ensure telemedicine did not negatively affect clinical outcomes, we compared in-person versus telemedicine differences in HbA1c, blood pressure, body mass index (BMI), and attendance.

**Results:**

The TUQ revealed that participants (N=19) found telemedicine useful and easy to use (4.9/5.0, 4.4/5.0, respectively) and with excellent interface (4.3/5.0), interaction (4.6/5.0), reliability (4.2/5.0), and satisfaction (4.4/5.0). There were no significant differences in clinical outcomes between arms: HbA1c (in-person: −0.60%, telemedicine: −0.52%, p=0.86), blood pressure (systolic: p=0.475, diastolic: p=0.683), weight (p=0.982), BMI (p=0.981), attendance (in-person: 75.44%, telemedicine: 70.12%, p=0.551).

**Conclusion:**

Provider telemedicine encounters in group visits are feasible and acceptable. This is a promising model to address provider limitations in group visits and increase access to care. Larger studies are needed to further evaluate these findings.

## Introduction

More than half of the world lacks access to essential health services [1,2]. Diabetes group visits, shared medical appointments that include education and medical evaluation, are cost-effective programs that have demonstrated increased healthcare access and improved clinical outcomes [3,4]. A four-year, multicenter randomized controlled trial (n=815) revealed that individuals receiving group visits significantly improved body mass index (BMI), fasting glucose, HbA1c, blood pressure, and cholesterol levels compared to those in usual care (p<0.001) [5]. Similarly, a systematic review of 26 diabetes group visit programs showed significant HbA1c reductions (-0.46%, 95% confidence interval −0.80% to −0.31%) [6].

However, ongoing shortages of primary care providers (PCPs) place group visits at risk [7]. Low- and middle-income countries have continued to face severe deficiencies [8]. There are 80 PCPs/100,000 US persons, which decreases to 68/100,000 in rural settings [9]. Though there are more PCPs/person in urban settings (84/100,000) [9], healthcare distribution is disproportionally lower for resource-poor populations and retention remains problematic [10].

COVID-19 has highlighted the value of telemedicine and the facilitation of health-related services via digital communication [11,12]. Yet, there are limited data exploring the use of telemedicine in group visits. A VA team from Hawaii conducted diabetes group visits with participants in Guam via telemedicine and found that intervention participants improved HbA1c levels compared to individuals in usual care (p=0.03) [13]. Another telemedicine group visit investigation of young adults with type 1 diabetes revealed fewer non-study office visits and hospitalizations of individuals in the program compared to those in usual care [14].

However these studies did not include low-income, uninsured, or minority individuals. Low-income settings often have local staff including Community Health Workers (CHWs), diabetes educators, and nurses available to conduct group visit education in-person [3]. These individuals offer great value to minority populations by enhancing healthcare systems’ understanding of cultural elements to care [15]. However, these settings are severely limited by lack of PCP availability [9,10].

Utilizing telemedicine for provider encounters while a local team conducts group visits in-person is a promising strategy to address provider limitations. The objective of this study was to evaluate the feasibility and acceptability of group visit provider encounters conducted via telemedicine while CHWs led the educational sections in-person for a low-income, Latino(a) population. Specifically, we compared in-person months 1–3 to telemedicine (videoconferencing) months 4–6. Outcomes included the Telehealth Usability Questionnaire (TUQ) [16] (primary) and in-person versus telemedicine clinical comparisons (HbA1c, blood pressure, BMI, attendance) to ensure there were no negative affects during the latter period. We hypothesized that telemedicine encounters would be feasible and acceptable and that these months would not negatively affect clinical outcomes.

## Materials and Methods

### Study Design

We conducted a prospective, feasibility and acceptability study of provider-patient telemedicine encounters in diabetes group visits at a 501(c)(3) community clinic that serves low-income (≤250% federal poverty level), uninsured individuals in Houston, Texas. The diabetes group visit program structure was based on our prior study [17]. Briefly, group visits met monthly for six months and consisted a clinical visit (vitals, labs, 1:1 physician encounter) and CHW-led education (large group and small group break-out sessions). The Institutional Review Board at Baylor College of Medicine approved the study (IRB H-40322). Written consent and signed group visit confidentiality forms were obtained from each participant.

### Provider-patient telemedicine encounters

ZOOM was the software platform. It provided end-to-end 256bit encrypted, secured, and HIPAA-compliant audio and video conferencing [18]. Participants first met with a provider (physician) in-person months 1–3 and then via telemedicine months 4–6. The provider encounter included reviewing laboratory data and home glucose logs (if applicable), medication titration and refills for diabetes, hypertension, and hyperlipidemia. During the in-person encounters, the provider met with the participant one-on-one in the same room.

During the telemedicine encounters, the provider met with participants from a remote site via video conferencing while study staff sat with the participant at the clinic for assistance e.g., holding up medication bottles, reading glucose logs, etc. A local physician was on-site at the clinic at all times should participants have needed to be seen in-person.

### Study Population

Inclusion criteria consisted included a documented diagnosis of type 2 diabetes (i.e., HbA1C ≥6.5), ability to understand Spanish, and self-identified as Latino(a). Individuals were excluded if their healthcare needs were too complex or not appropriate for a group setting (i.e., pregnancy). Potential participants were identified primarily by the clinic database. Study staff called the eligible participants, explained the study, and, if interested, invited them to an orientation where they obtained baseline data and written consents [17].

### Measures

Feasibility and acceptability were measured by the TUQ (primary outcome). The TUQ consists of 21 questions ranked on a fivepoint Likert scale (definitely disagree, disagree, neutral, agree, and definitely agree) and divided into six sections: usefulness (3 questions), ease of use (3 questions), interface and interaction quality (4 questions), reliability (3 questions), and satisfaction and future use (4 questions). Each of these variables has demonstrated good to excellent internal consistency (standardized Cronbach coefficient alpha 0.81–0.93) and strong content validity and reliability [16]. In addition, three open-ended, free-text questions were added at the end of TUQ to provide descriptive data of participant likes, dislikes, and items they would like to change about the telemedicine process.

Due to a limited literacy in low-income settings and minority populations [19], surveys were read aloud by a native Spanish speaker and assistance for writing was provided as needed. The TUQ was translated from English to Spanish using ISO 17100:2015 compliant GTS Translation Service [20].

We also evaluated eight focal areas of feasibility: acceptability (how participates reacted to telemedicine), adaptation (changing program (if applicable)), demand for intervention (attendance), expansion (potential success within a different population or setting), limited-efficacy testing (i.e., TUQ, clinical outcomes), implementation (likelihood telemedicine could be conducted as proposed), integration (level of change needed to initiate telemedicine), and practicality (extent telemedicine could be delivered) [21]. Further, to evaluate if participants had adverse effects associated with telemedicine, we compared the baseline to 6-month change of HbA1c, blood pressure, and BMI.

### Statistical Analyses

SigmaPlot version 13.0 was used for statistical analysis. Descriptive statistics, including mean and standard deviation was performed on the TUQ scores, HbA1c, weight, BMI, and blood pressures. A paired t-test was used for normally distributed data. Wilcoxon Signed Rank test was used to evaluate distributions that failed normality test (Shapiro-Wilk). TUQ missing data was omitted from the analysis. Clinical missing data was handled by last observation carried forward. Analysis was intention to treat. Statistical significance was set at p<0.05 was considered statistically significant. This was a feasibility and acceptability study and not powered for statistical significance [21].

## Results

Baseline characteristics of the participants are illustrated in Table 1. Our sample consisted of slightly more females (52.6%), and the average age was 52 years (range 27–63 years). The most common work was domestic (52.6%) followed by manual labor (e.g., construction, landscaping) (36.9%) and food service (10.5%). The mean time since diagnosis of diabetes was less than 10 years (mean 8.69). Baseline clinical data showed mean HbA1c levels were uncontrolled (8.46%) and BMI averaged in the obese range (37.77 kg/m^2^). Other baseline clinical levels were near-normal: LDL cholesterol (101.18 mg/dL), triglycerides (186.89 mg/dL), and blood pressure (133.50/77.68).

### Telehealth Usability Questionnaire

The TUQ resulted in systematic positive findings (Table 2). Participants reported high levels in regards to its usefulness (4.9/5.0), ease of use (4.4/5.0), interface (4.3/5.0), interaction (4.6/5.0), reliability (4.2/5.0), and their satisfaction (4.4/5.0). Descriptive data revealed that participants valued clear visualization and real-time conversations without interruption the most.

### Secondary Outcomes

Participants improved glycemic control during the six-month diabetes group visits (HbA1c: 8.46 to 7.86%, p=0.12), which had a moderate effect size (49.7). Within group comparisons revealed that HbA1c improvements were comparable during the in-person months 1–3 versus the telemedicine months 4–6 (-0.60% (effect size 25.0) vs. −0.52% (effect size 24.0), respectively). Similarly, in-person versus telemedicine blood pressure, BMI, and weight changes were not significantly different. Blood pressure trends favored the telemedicine more than the in-person months: (systolic: 4.31 vs. −2.53 mmHg (p=0.475), diastolic: 2.63 vs. −1.32 mmHg (p=0.683), respectively). Attendance was comparable during in-person and telemedicine encounters (75.4% vs. 70.2%, respectively, p=0.551) (Table 3).

## Discussion

This study evaluated patient-provider telemedicine encounters during group visits and found that they were feasible and acceptable as evidenced by systematic positive findings on the TUQ and no negative clinical impact during the virtual months. COVID-19 has highlighted telemedicine’s ability to facilitate healthcare [11,12]. Telemedicine could greatly improve diabetes group visit scalability and sustainability. However, there is a limited data demonstrating its use in these important programs [13,14] and no reported investigations in low-income, minority populations prior to the current study.

Numerous studies have demonstrated that diabetes group visits are valuable in improving health outcomes and reducing disparities [3,4,6]. Utilizing telemedicine for group visits has the potential to markedly increase access to care, which is becoming increasingly important with ongoing primary care deficiencies [7]. The heightening shortage of primary care providers that disproportionately affects underserved communities has made access to healthcare a pressing issue [8–10]. The COVID-19 pandemic has underscored disparities amongst low-income minorities and the value of telemedicine [11,12]. New and innovative ways are critical to reach communities. Healthcare practitioners are a vital piece of group visits as they provide medications review, titration, and refills [22–24]. This telemedicine modality has the ability to enable providers to care for individuals across expansive geographical borders while locally trained individuals, such as CHWs, provide education on-site. Maintaining local staff educators is especially beneficial in addressing cultural barriers and individualizing needs, particularly in low-income and minority populations [15].

There are several important points related to eight feasibility focal areas: acceptability, adaptation, demand, expansion, limited-efficacy testing, implementation, integration, practicality [21]. The program was acceptable as measured by patient satisfaction (TUQ), particularly the intent to continue use (mean 4.4/5), and by clinical data. Limited-efficacy testing was demonstrated by positive TUQ and noninferior clinical outcomes. There was a consistent demand for the program as documented by the attendance. There was a slight, nonsignificant drop (75.4% to 70.2%, p=0.551) from months 1–3 to 4–6 but this was more likely due to the longevity of the class rather than a reflection of the telemedicine encounters. Prior studies have also supported the demand of telemedicine in diabetes care; A 12-month randomized controlled trial comparing telemedicine diabetes self-management education revealed greater HbA1c reductions in the intervention group (9.4 to 8.2% vs. 8.8 to 8.6%, respectively), which continued to improve for 24-months [25].

A key part of integration, and thereby expansion, is a needs and asset assessment [26]. For example, the healthcare site’s technology structure and software platforms, which are widely variable particularly within low-income settings, need to be examined to determine what structures are in place and will need to be added. Implementation and practicality success in the current study was supported by the software program and server (ZOOM) that has been used extensively worldwide and is encrypted for patient safety [18,27]. The Internet was occasionally unreliable, causing the video-conference to stall or disconnect entirely, which may be reflected in some of the lower TUQ scores. Conducting the study over a reliable Internet or a physical connection via ethernet port can mitigate this issue.

To evaluate expansion, further investigations are needed with larger sample sizes and in other settings. This study is limited by size and in one locale but it provides foundational data to expand to larger, more diverse areas. Also, since all patients spoke Spanish but not all study staff members were bilingual, this may have increased telemedicine encounter time and altered TUQ outcomes. We observed that when native speakers interacted with the participants, encounters were smoother and the encounter times decreased.

## Conclusion

These findings provide preliminary data of a novel intervention of patient-provider telemedicine encounters in group visits that is promising to increase access to care and sustainability of these valuable programs. These findings are particularly important with the ongoing shortages of primary care providers worldwide and critical needs to increase access to care in vulnerable populations. Larger multi-center studies are warranted to evaluate the expansion of these findings.

## Figures and Tables

**Table 1:** Baseline participant characteristics (N=19).

	Biographical Data	n (%) or mean (SD)
**Sex**	Male	9 (47.4)
	Female	10 (52.6)
**Age (years)**		51.9 (8.65)
**Work**	Construction/landscaping	7 (36.9)
	Domestic (homemaker, housekeeping)	10 (52.6)
	Food service	2 (10.5)
**Biochemical Data**		mean (SD)
**Time since diabetes diagnosis (years)**		8.56 (7.43)
**Hemoglobin A1c (%)**		8.46 (2.41)
**Cholesterol (mg/dL)**	Total	172.56 (31.96)
	HDL	45.06 (11.25)
	LDL	101.18 (31.25)
	Triglycerides	186.89 (135.73)
**Blood Pressure (mmHg)**	Systolic blood pressure	133.74 (15.13)
	Diastolic blood pressure	77.68 (11.67)
**Body Mass Index (kg/m^2^)**		37.77 (10.03)
**Weight (kg)**		82.48 (42.02)

**Table 2:** The number of participant (N=19) responses on the 21-question Telehealth Usability Questionnaire stratified by category and question.

Section	Question (n)	Highly Disagree	Disagree	Neutral	Agree	Highly Agree	Number of Respondents	Average	Average of Section
**Usefulness**	1				6	9	15	4.60	4.49
	2			1	8	8	17	4.41	
	3			1	8	9	18	4.44	
**Ease of Use**	4		1		8	9	18	4.39	4.44
	5		1		6	11	18	4.50	
	6				10	8	18	4.44	
**Interface Quality**	7		1		11	5	17	4.18	4.29
	8		1		8	8	17	4.35	
	9		1		9	7	17	4.29	
	10		1		7	7	15	4.33	
**Interaction Quality**	11				5	12	17	4.71	4.55
	12				7	10	17	4.59	
	13		1	1	6	10	18	4.39	
	14			1	6	10	17	4.53	
**Reliability**	15		2	2	6	8	18	4.11	4.17
	16		1	2	8	6	17	4.12	
	17		2		4	8	14	4.29	
**Satisfaction, Future Use**	18		1		8	9	18	4.39	4.40
	19		1	1	7	8	17	4.29	
	20		1		7	9	17	4.41	
			1		6	11	18	4.50	

**Table 3:** Participant clinical and attendance outcomes (N=19).

Variable		Mean Change (SD or %)	p-value*
**HbA1c (%)**	Baseline to 3-months	−0.60 (1.42)	0.86
	3- to 6-months	−0.52 (1.06)	
	Baseline to 6-month	−1.12 (1.50)	
**Weight (kg)**	Baseline to 3-months	−0.44 (2.15)	0.982
	3- to 6-months	−0.19 (2.63)	
	Baseline to 6-month	−0.63 (2.93)	
**BMI (kg/m^2^)**	Baseline to 3-months	−0.17 (0.81)	0.981
	3- to 6-months	−0.08 (1.02)	
	Baseline to 6-month	−0.26 (1.10)	
**Systolic blood pressure (mmHg)**	Baseline to 3-months	4.31 (15.10)	0.475
	3- to 6-months	−2.53 (12.07)	
	Baseline to 6-month	−1.79 (13.91)	
**Diastolic blood pressure (mmHg)**	Baseline to 3-months	2.63 (10.26)	0.683
	3- to 6-months	−1.32 (9.17)	
	Baseline to 6-month	1.32 (9.33)	
**Attendance**	Baseline to 3-months (total classes attended)	43 (75.44%)	0.551
	3- to 6-months	40 (70.18%)	
	Baseline to 6-month	83 (72.81%)	

## References

[1] Levesque JF, Harris MF, Russell G. Patient-centred access to health care: conceptualising access at the interface of health systems and populations. Int J Equity Health. Mar 11 2013;12:18. 23496984 10.1186/1475-9276-12-18 PMC3610159

[2] World Health Organization. Tracking universal health coverage: 2017 Global Monitoring Report 2017; https://apps.who.int/iris/bitstream/handle/10665/259817/9789241513555-eng.pdf. Last accessed July 29, 2020.

[3] Vaughan EM, Johnston CA, Arlinghaus KR, Hyman DJ, Foreyt JP. A Narrative Review of Diabetes Group Visits in Low-Income and Underserved Settings. Curr Diabetes Rev. 2019;15(5):372–381. 30421682 10.2174/1573399814666181112145910 PMC6511502

[4] Housden LM, Wong ST. Using Group Medical Visits With Those Who Have Diabetes: Examining the Evidence. Curr Diab Rep. Dec 2016;16(12):134. 27909958 10.1007/s11892-016-0817-4

[5] Trento M, Gamba S, Gentile L, Rethink Organization to iMprove Education and Outcomes (ROMEO): a multicenter randomized trial of lifestyle intervention by group care to manage type 2 diabetes. Diabetes Care. Apr 2010;33(4):745 747. 10.2337/dc09-2024 PMC2845019 20103547

[6] Housden L, Wong ST, Dawes M. Effectiveness of group medical visits for improving diabetes care: a systematic review and meta-analysis. CMAJ. Sep 17 2013;185(13):E635–644. 23939218 10.1503/cmaj.130053 PMC3778483

[7] Frisch S. The primary care physician shortage. BMJ. Nov 4 2013;347:f6559. 24188903 10.1136/bmj.f6559

[8] Bitton A, Fifield J, Ratcliffe H, Primary healthcare system performance in low-income and middle-income countries: a scoping review of the evidence from 2010 to 2017. BMJ Glob Health. 2019;4(Suppl 8):e001551. 10.1136/bmjgh-2019-001551 PMC6703296 31478028

[9] Petterson SM, Phillips RL Jr, Bazemore AW, Koinis GT. Unequal distribution of the U.S. primary care workforce. Am Fam Physician. Jun 1 2013;87(11):Online. 23939507

[10] DeAngelis CD. Commitment to care for the community. JAMA. May 13 2009;301(18):1929–1930. 19436023 10.1001/jama.2009.642

[11] Vidal-Alaball J, Acosta-Roja R, Pastor Hernandez N, Telemedicine in the face of the COVID-19 pandemic. Aten Primaria. Jun - Jul 2020;52(6):418–422. 32402477 10.1016/j.aprim.2020.04.003 PMC7164871

[12] Smith AC, Thomas E, Snoswell CL, Telehealth for global emergencies: Implications for coronavirus disease 2019 (COVID-19). J Telemed Telecare. Jun 2020;26(5):309–313. 32196391 10.1177/1357633X20916567 PMC7140977

[13] Tokuda L, Lorenzo L, Theriault A, The utilization of video-conference shared medical appointments in rural diabetes care. Int J Med Inform. Sep 2016;93:34–41. 27435945 10.1016/j.ijmedinf.2016.05.007

[14] Wan W, Nathan AG, Skandari MR, Cost-effectiveness of Shared Telemedicine Appointments in Young Adults With T1D: CoYoT1 Trial. Diabetes Care. Aug 2019;42(8):1589 1592. 31189564 10.2337/dc19-0363 PMC6647044

[15] Gampa V, Smith C, Muskett O, Cultural elements underlying the community health representative - client relationship on Navajo Nation. BMC Health Serv Res. Jan 9 2017;17(1):19. 28069014 10.1186/s12913-016-1956-7 PMC5223387

[16] Parmanto B, Lewis AN Jr, Graham KM, Bertolet MH. Development of the Telehealth Usability Questionnaire (TUQ). Int J Telerehabil. Spring 2016;8(1):3–10. 10.5195/ijt.2016.6196 PMC4985278 27563386

[17] Vaughan EM, Hyman DH, Naik AD, Samson SL, Razjouyan J, Foreyt JP. A Telehealth-supported, Integrated care with CHWs, and MEdication-access (TIME) program for diabetes improves HbA1c: A randomized clinical trial. Journal of General Internal Medicine. 2020; 10.1007/s11606-02006017-4. PMC7878600 32700217

[18] ZOOM. Zoom for Healthcare. 2020; https://zoom.us/healthcare. Last accessed July 8, 2020.

[19] White RO, Wolff K, Cavanaugh KL, Rothman R. Addressing health literacy and numeracy to improve diabetes education and care. Diabetes Spectr 2011;23(4):238–243. 10.2337/diaspect.23.4.238 PMC3032499 21297890

[20] Medical Translation Agency. Medical Translation Services. 2019; https://www.gts-translation.com/services/medical_translation_services/. Last accessed July 29, 2020.

[21] Bowen DJ, Kreuter M, Spring B, How we design feasibility studies. Am J Prev Med. 2009;36(5):452–457. 19362699 10.1016/j.amepre.2009.02.002 PMC2859314

[22] Brown MT, Bussell JK. Medication adherence: WHO cares? Mayo Clin Proc. Apr 2011;86(4):304–314. 21389250 10.4065/mcp.2010.0575 PMC3068890

[23] Kleinsinger F. The Unmet Challenge of Medication Nonadherence. Perm J. 2018;22:18–033. 10.7812/TPP/18-033 PMC6045499 30005722

[24] Polonsky WH, Henry RR. Poor medication adherence in type 2 diabetes: recognizing the scope of the problem and its key contributors. Patient Prefer Adherence. 2016;10:1299–1307. 27524885 10.2147/PPA.S106821 PMC4966497

[25] Davis RM, Hitch AD, Salaam MM, Herman WH, ZimmerGaller IE, Mayer-Davis EJ. TeleHealth improves diabetes self-management in an underserved community: diabetes TeleCare. Diabetes Care. Aug 2010;33(8):1712–1717. 20484125 10.2337/dc09-1919 PMC2909047

[26] Sharpe PA, Greaney ML, Lee PR, Royce SW. Assets-oriented community assessment. Public Health Rep. Mar-Jun 2000;115(2–3):205–211. 10968755 10.1093/phr/115.2.205 PMC1308712

[27] De Witt Jansen B, Brazil K, Passmore P, Evaluation of the impact of telementoring using ECHO(c) technology on healthcare professionals’ knowledge and self-efficacy in assessing and managing pain for people with advanced dementia nearing the end of life. BMC Health Serv Res. Apr 2 2018;18(1):228. 29606132 10.1186/s12913-018-3032-y PMC5879835

